# Characteristics of Oat and Buckwheat Malt Grains for Use in the Production of Fermented Foods

**DOI:** 10.3390/foods12203747

**Published:** 2023-10-12

**Authors:** Agnieszka Salamon, Hanna Kowalska, Anna Ignaczak, Agata Marzec, Jolanta Kowalska, Anna Szafrańska

**Affiliations:** 1 Department of Grain Processing and Bakery, Prof. Wacław Dąbrowski Institute of Agricultural and Food Biotechnology—State Research Institute, 36 Rakowiecka St., 02-532 Warsaw, Poland; anna.szafranska@ibprs.pl; 2 Department of Food Engineering and Process Management, Institute of Food Sciences, Warsaw University of Life Sciences, 159c Nowoursynowska St., 02-776 Warsaw, Poland; anna_ignaczak@sggw.edu.pl (A.I.); agata_marzec@sggw.edu.pl (A.M.); jolanta_kowalska@sggw.edu.pl (J.K.)

**Keywords:** oat and buckwheat malt, wort, technological quality, antioxidant properties, fermented food

## Abstract

Malted gluten-free cereal grains and pseudo-cereals are interesting raw materials for producing fermented foods. The aim of the work was to assess selected technological quality characteristics and antioxidant properties of special malts in terms of use in the production of fermented foods. The research material consisted of malts made from oat, buckwheat, and brewing barley. Malting was performed on a microtechnical scale according to the standard scheme for brewing barley grain. The basic quality parameters of cereal grains obtained malts, and laboratory wort were assessed according to methods applicable in brewing. Atypical brewing malts were characterized by parameters such as malt extractability, protein solubilization, diastatic force, mash filtration time, and wort viscosity. The best results, comparable to barley malt, were obtained for naked oat malt. Malted buckwheat grains turned out to be the least biochemically modified, although their use in the production of beer and/or other fermented beverages is supported by the high content of bioactive substances and antioxidant potential. As the malting process of cereal plants improves their antioxidant properties and increases their nutritional value, oat and buckwheat malts can be successfully used to produce gluten-free fermented beverages or as an addition to fermented products, e.g., in baking and confectionery.

## 1. Introduction

Malting is a technological process aimed at modifying brewing barley and wheat grain to obtain traditional malts used in beer production technology. During the steeping of grain, and then germination and kilning, amylolytic, proteolytic, and cytolytic enzymes are activated as a result of the decomposition of proteins and starch, the endosperm structure is loosened, and the malt gains appropriate sensory characteristics [1,2]. However, nowadays, beers made with the addition of other ingredients or based solely on ingredients other than barley malt are gaining more and more popularity. In the world, beers are produced from malts obtained from almost every cereal grown in a given geographical area [3,4]. Recently, legume seeds have also been malted [5,6]. The process of malting grain other than brewing barley remains a great challenge for malt producers because process conditions must be optimized to obtain malt with qualitative characteristics similar to barley or wheat malt [7,8,9]. Used in brewing, atypical grains, malted or unmalted, can give beer completely new organoleptic features and other positive properties [6,10,11,12].

Breweries, in response to the growing needs of consumers who are more open to beer innovations, want to meet their expectations and introduce new categories of beers to the market, for the production of which they use not only traditional malts, i.e., barley and wheat malt [5,13]. The constantly growing interest of society in food with health-promoting properties and for special nutritional purposes contributes to drawing attention to gluten-free cereals and pseudo-cereals [14,15,16,17].

Gluten is water-insoluble storage proteins (prolamins) found in wheat, barley, and rye, and avenins found in oats that can cause celiac disease in genetically susceptible individuals manifested by an inappropriate immune system response to gluten ingestion. Compared to wheat prolamins (80–85%), oat avenins account for only 10–15% of the total protein content [8,18]. The presence of gluten in the diet of people with celiac disease leads to malabsorption of nutrients and related health problems. Celiac disease and gluten-dependent diseases (e.g., gluten allergy, non-celiac gluten sensitivity) are constantly increasing and currently affect about 1% of the adult European population [19,20]. The only way to treat celiac disease is to completely eliminate gluten from your diet.

The oat and buckwheat, next to basic cereals, are among Poland’s most frequently cultivated cereals. They are a rich source of nutrients and biologically active substances, such as non-starch polysaccharides, B vitamins, minerals, and bioactive substances [8,21,22]. Buckwheat does not contain gluten [23,24], and many years of clinical research show that most people with gluten intolerance can include oats and oat products in their diet without negative health effects [25,26,27]. Controlled oat cultivation, from sowing to processing, is a prerequisite for excluding its contamination with gluten cereals. In EU countries, the USA, and Canada, oat products are considered gluten-free if the gluten level does not exceed 20 mg/kg [23]. Nevertheless, Comino et al. [27] point out that the immunogenicity of oats depends on the cultivar. Hartmann et al. [18] proved that proteinases of sprouting cereal grains can significantly reduce the level of gluten protein fraction (prolamins) as a result of the hydrolysis of peptide bonds.

The selection of malting and mashing parameters for atypical brewing malts is not easy due to the structure and chemical composition of the grain, the pool of enzymes, and the optimal conditions for their operation depending on the properties of the raw material [8,28,29,30]. Beer may not be an essential component of the diet, but the presence of biologically active substances in beer, with its moderate consumption, seems to indicate the potential therapeutic properties of this beverage [31,32,33]. In patients with celiac disease, beer consumption can improve the quality of life, especially since people diagnosed in adulthood already have developed eating habits that may be difficult to change [19].

Gluten-free beers are produced using two methods [34]. The first consists of using classic brewing raw materials and removing gluten during the process. Dostálek et al. [35] found a decrease in gluten levels during mashing, fermentation, and beer stabilization. Reducing the amount of gluten in beer can also be achieved enzymatically [15,19,36]. In addition, beers currently produced with the addition of gluten-free unmalted raw materials, e.g., corn, rice, and carbohydrate syrups, generally contain lower amounts of gluten [34]. The second way of producing gluten-free beer is to use gluten-free malt produced from cereals, e.g., oats from controlled crops, rice, millet, or pseudo-cereals, e.g., buckwheat, amaranth, sorghum, quinoa. Therefore, oat and buckwheat malt grains can be a valuable raw material for the production of innovative functional beverages and an attractive alternative to the specialty beer market [30,37,38].

The production of gluten-free beer from 100% oat or buckwheat malt has been attempted with varying success. The obtained products had more or less promising flavor and aroma profiles [11,12,16,28,30,39]. In the study by Klose et al. [40], sensory evaluation of oat beer revealed an intense flavor similar to blueberries, raspberries, or yogurt. It contained lower amounts of volatile fermentation products and carbonyl compounds than in barley beer. In turn, top-fermented buckwheat beer obtained by Phiarais et al. [16] was similar to typical wheat beer in terms of alcohol content, degree of attenuation, and pH value. The concentration of fusel alcohol was comparable to the normative values for top-fermented beers. Gas chromatography (GC) analysis of the resultant beer revealed a high level of ethyl caprinate (coconut flavor) and lauric acid (fatty odor). Sensory analysis indicated that the buckwheat beer was acceptable with regard to odor, purity of taste, mouthfeel, tingling, and bitterness.

The use of oat and buckwheat malt for the production of gluten-free beers or cereal-based fermented beverages may contribute to the so-called “new-wave” products with interesting sensory characteristics, which may find recipients not only among patients with celiac disease and gluten-dependent diseases but also among people looking for various types of market novelties.

The aim of the study was to assess the characteristics of technological quality and antioxidant potential of oat and buckwheat malt for use in the production of gluten-free beer and other fermented products.

## 2. Materials and Methods

### 2.1. Cereals

The research material consisted of cereals and pseudo-cereals collected in the 2012 harvest year in Poland. Two samples of hulled yellow oats (YO1 and YO2) and one sample of hulled brown oats (BO), naked oats (NO), and buckwheat (Bw) were used for malting (Table 1). Spring brewery barley (B) was the reference sample. Oat and barley samples for testing were made available by Strzelce Plant Breeding Ltd. PBAI Group (Strzelce, Poland), and the buckwheat sample was purchased from Małopolska Plant Breeding Ltd. (Cracow, Poland).

Before the micro-malting process, the brewing barley was sorted on a 2.5 mm sieve in order to separate the full grain, i.e., the fraction most desirable for brewing purposes. In the case of oats and buckwheat grains, grain sorting on the barley sorter was abandoned, due to the smaller size of the oat grains compared to barley and the different shape of buckwheat grains.

### 2.2. Cereals Characteristics

Before the micro-malting process, the quality of barley, oat, and buckwheat grains was determined by basic parameters characterizing them, such as the sieving test using Sortimat sieves (Pfeuffer, Kitzingen, Germany) according to method 3.11.1 [41], germinative energy after 72 and 120 h using the Schönfeld method according to method 3.6.3 [41], the moisture content by the drying oven method according to method 3.2 [41], the total protein content (N × 6.25) by the Kjeldahl method using a Kjeltec System (Foss Tecator, Hoganas, Sweden) according to method 3.3.1 [41] and the fat content by the Soxhlet method using an apparatus Soxtec 20483 (Foss Tecator, Hoganas, Sweden) [42]. In addition, in selected grain samples, the percentage of husk content by the gravimetric method after manual dehulling and the diastatic power by the iodometric titration method [43] were determined. The results of diastatic power were expressed in °WK (Windisch–Kolbach units). The determinations were carried out in duplicate.

### 2.3. Micro-Malting Procedure

The samples were malted on a microtechnical scale at the Prof. Wacław Dąbrowski Institute of Agricultural and Food Biotechnology—State Research Institute using as a standard scheme for brewing barley according to method 1.5.3 [44]. Steeping with aeration was performed until the water content in the samples reached 45% according to the following: 1st day—5 h underwater and 19 h air rest, 2nd day—4 h underwater and 20 h air rest, 3rd day—water spray to obtain grain moisture in the air phase. The steeping and germination of grain was carried out at an air temperature of 14 ± 1 °C and relative humidity above 95%. Samples for kilning were taken after 5 days of germination. The malting was then terminated using an increasing kilning regime of 50 °C—16 h, 60 °C—1 h, 70 °C—1 h, and 80 °C—5 h for a total of 23 h. After cooling the malt, the rootlets were removed. The dried samples were stored at ambient temperature for further analysis.

The malting yield (My), i.e., the percentage ratio of the amount of malt produced to the amount of grains (barley, oat, and buckwheat) used, was calculated on a dry matter basis. Malting of grain samples was carried out in two parallel repetitions.

### 2.4. Malt Characteristics

The malts were finely ground into flour (2.0 mm) using a DLFU disc mill (Bühner-Miag, Braunschweig, Germany) according to method 3.1.4.2.1 [44]. In malt flour, the following parameters were determined: the moisture content by the drying oven method using a dryer Digitheat (J.P. Selecta, Barcelona, Spain) according to method 4.2 [41], the total nitrogen (TN) content by the Kjeldahl method using a Kjeltec System (Foss Tecator, Hoganas, Sweden) and converted into protein content (N × 6.25) according to method 3.1.4.5.1.1 [41], the diastatic power (DP) by the iodometric titration method [43] and the beta-glucan content by the spectrophotometric method after prior enzymatic hydrolysis using the (1-3) (1-4) Beta-D-Glucan Kit (Megazyme, Bray, Wicklow, Ireland) on a Beckman DU-530 spectrophotometer (Beckman, Wycombe, UK) according to method 4.16.1 [41]. The content of dimethyl sulfide precursors (DMS-P) in malts was determined by gas chromatography with flame photometric detection (GC/FPD) using an HP 6890 gas chromatograph (Hewlett Packard, Wilmington, Germany) with an HP 7694E headspace autosampler (Hewlett Packard, Wilmington, Germany) and an HP-FFAP capillary column (30 m × 0.53 mm × 1 µm) (Agilent Technologies, Santa Clara, CA, USA) according to method 3.1.4.17 [44]. The determinations were carried out in duplicate.

### 2.5. Malt Mashing

The process of malt mashing to obtain laboratory wort (so-called congress wort) was performed according to the standard mashing scheme for barley malt (i.e., 45 °C for 30 min, 1 °C/min, 70 °C for 60 min) using an LB Electronic mashing device (Lochner Labor + Technik, Berching, Germany) according to method 3.1.4.2.2 [44]. The saccharification time using the iodine test according to method 3.1.4.2.4 [44] and the time of wort filtration according to method 3.1.4.2.5 [44] were determined. The determinations were carried out in duplicate.

### 2.6. Congress Wort Characteristics

In samples of laboratory wort obtained from malt, the following parameters were determined: the extract content (W-Ex) by the oscillometric method using a DMA 5000 oscillating density meter (Anton Paar, Graz, Austria) and it converted to the extract content in the dry matter of malt, i.e., malt extractivity (M-Ex) according to method 3.1.4.2.2 [44], the soluble nitrogen (SN) by the Kjeldahl method using a Kjeltec System (Foss Tecator, Hoganas, Sweden) and the Kolbach Index (KI) were calculated according to method 3.1.4.5.2.1 [44], pH according to method 3.1.4.2.7 [44], the free amino nitrogen (FAN) content by the spectrophotometric method using a Beckman DU-530 spectrophotometer (Beckman, Wycombe, UK) according to method 3.1.4.5.5.1 [44], the limit of attenuation (LA) of congress wort by the 24 h fermentation with beer yeast according to method 3.1.4.10.1.3 [44], and the viscosity (V) of wort using a CPE-40 spindle and a DV-II+ viscometer (Brookfield, Middleborough, MA, USA) according to method 8.4 [41]. The fermentable carbohydrates (FC) content (i.e., fructose, glucose, maltotriose, and disaccharides as the sum of maltose and sucrose) was determined by high-performance liquid chromatography method (HPLC) according to method 8.7 [41] using a Waters HPLC System (Waters, Milford, MA, USA). The system was equipped with a Waters 2695 separation module (pump, autosampler, and degasser), a Waters 2414 refractive index detector, a thermostat for columns, and the Sugar-Pak I column (300 mm × 6.5 mm) with a guard column (Waters, Milford, MA, USA). The determinations were carried out in duplicate.

### 2.7. Polyphenol Content and Antioxidant Activity of Malt

#### 2.7.1. Extract Preparation

To obtain the extracts, samples were ground into flour (2.0 mm) using a DLFU disc mill (Bühner-Miag, Braunschweig, Germany) according to method 3.1.4.2.1 [44]. A quantity of 25 mL 80% (*v/v*) of ethyl alcohol solution was added to the weighed sample (approx. 2.0 g). The samples were homogenized in an ULTRA-TURRAX T10 basic homogenizer (IKA-WERKE, Staufen, Germany) for 30 s at 13,500 rpm. The homogenates were kept in the dark for 30 min at room temperature. Then, the obtained homogenate was centrifuged in a SIGMA 4-15 laboratory centrifuge (Sartorius, Getynga, Germany) for 3 min at 10,000 rpm. Two extracts were made for each sample.

#### 2.7.2. Determination of Total Polyphenols Content with Folin–Ciocalteu Reagent

The total polyphenol content (TPC) was determined by the spectrophotometric method using a UV–vis Heλios γ spectrophotometer (Thermo Electron Corporation, Waltham, MA, USA) with the Folin–Ciocalteu reagent (ChemPur, Piekary Śląskie, Poland), which consisted of a colored reaction of polyphenolic compounds with this reagent [45]. In a glass test tube, 4.92 mL of distilled water, 0.18 mL of the extract, and 0.3 mL of Folin–Ciocalteu reagent were mixed. After 3 min, 0.6 mL of supersaturated sodium carbonate was added. The mixtures were incubated in the dark for 1 h at 25 °C, and then their absorbance was measured at λ = 750 nm. The total polyphenol content was calculated based on the calibration curve prepared for gallic acid (GA; POL-AURA, Zawroty, Morąg, Poland). The results were expressed in mg GA per 100 g of dry matter (d.m.) of the malt. The determinations were carried out in duplicate.

#### 2.7.3. Determination of Antioxidant Activity by DPPH Assay

The antioxidant activity (AA) was determined using the spectrophotometric method with the DPPH radical (1,1-diphenyl-2-picrylhydrazyl; TCI, Tokyo, Japan) using a Heλios γ UV–vis spectrophotometer (Thermo Electron Corporation, Waltham, MA, USA) based on the method of Urbańska et al. [45]. For the preparation of samples, 2.4 mL of DPPH radical solution (75 μM) in 80% (*v*/*v*) ethyl alcohol was used and 100 μL of 80% (*v*/*v*) ethyl alcohol extract of the samples was added. The samples were mixed and incubated at room temperature for 30 min in the dark. After this time, the absorbance was measured at the wavelength λ = 515 nm against the blank. The 80% (*v*/*v*) ethyl alcohol solution and DPPH solution were collected for the control sample. The blank was 80% (*v*/*v*) ethyl alcohol. The antioxidant activity (scavenging capacity) of the DPPH radical (% inhibition) was calculated:

% inhibition = [(A_0_ − A_1_)/A_0_] · 100 (1)

where:

A_1_—absorbance of the DPPH radical with ethyl alcohol extract from the sample;
A_0_—absorbance of the DPPH radical with ethyl alcohol (control sample).

The antioxidant activity (AA) based on the DPPH free radical scavenging ability of the extract was expressed as µM Trolox (Sigma-Aldrich, Buchs, Switzerland) per 1 g of dry matter (d.m.) of the malt. All determinations were performed at least in duplicate.

### 2.8. Carotenoids and Chlorophylls Content in Malt

The carotenoids and chlorophylls content in the sample of malts were measured using a BECKMAN DU-530 spectrophotometer (Beckman, Wycombe, UK). The samples were ground in an IKA A11 basic analytical grinder (IKA-WERKE, Staufen, Germany), weighed (approx. 4.0 g), and added to 25 mL of 80% (*v*/*v*) to obtain an acetone solution extract. The samples were homogenized in an ULTRA-TURRAX T25 homogenizer (IKA-WERKE, Staufen, Germany) for 30 s at 13,500 rpm. The homogenates were kept in the dark for 30 min at room temperature. Then, the obtained homogenate was centrifuged in an MPW 375 laboratory centrifuge (MPW-Med-Instruments, Warsaw, Poland) for 3 min at 10,000 rpm. Two extracts were made for each sample. The measurements were made for chlorophyll A at wavelengths λ = 663 nm, for chlorophyll B at λ = 647 nm, and at λ = 470 nm for carotenoids with the blank, which was 80% (*v*/*v*) acetone solution. When the measured absorbance of the sample was greater than 0.900 in value, the sample was diluted with an 80% (*v*/*v*) acetone solution. The determination was performed in duplicate. 

The content of carotenoids and chlorophylls pigments in the acetone extract was calculated from the equations below [46]:

C_C_ = (1000 · A_470_ − 1.82 · C_A_ − 85.02 · C_B_)/198 (2)

C_A_ = 12.25 · A_663_ − 2.79 · A_647_ (3)

C_B_ = 21.50 · A_647_ − 5.10 · A_663_ (4)

C_A+B_ = 7.15 · A_663_ + 18.71 · A_467_ (5)

where:

C_C_—carotenoids content in acetone extract, (μg/mL);
C_A_—chlorophyll content A in acetone extract, (μg/mL);
C_B_—chlorophyll B content in the acetone extract, (μg/mL);
C_A+B_—chlorophylls (A + B) content in the acetone extract, (μg/mL);
A_663_—absorbance of acetone extract measured at the wavelength λ = 663 nm;
A_647_—absorbance of acetone extract measured at the wavelength of λ = 647 nm;
A_470_—absorbance of acetone extract measured at the wavelength λ = 470 nm.

The content of carotenoids and chlorophylls in the malt samples was calculated in µg per 1 g of dry matter (d.m.). All determinations were performed at least in duplicate.

### 2.9. Statistical Analysis

The statistical analysis of the obtained results was performed with the use of Microsoft Excel and STATISTICA 13 PL programs (StatSoft, Cracow, Poland). A one-factor analysis of variance and Tukey’s HSD test were performed to determine homogeneous groups (post hoc test), and Pearson’s correlation was performed to investigate the relationship between the selected indicators. Calculations were made at the significance level of *p* = 0.05. In addition, the results were subjected to principal component analysis (PCA) and cluster analysis.

## 3. Results and Discussion

### 3.1. Cereals Characteristics

The results of assessing the suitability for malting samples of brewing barley, hulled and naked oats, and buckwheat are presented in Table 2.

The grains of the tested samples were characterized by acceptable germinative energy (Table 2). Their germination capacity after 120 h was above 78%, except for buckwheat grains which was 67%. The low germination capacity of buckwheat may indicate insufficient maturity or heterogeneity of the grain. In the studies of Pawłowska et al. [48], the germination energy of Sławko oats cultivar was approx. 83.1%. 

The penetration of water into the grain at the steeping stage depends not only on the time and temperature of the process but also on the type of grain, kernel size, the presence of husk, and the moisture and protein content in the grain. Water penetrates the embryo and then the endosperm and aleurone layer of the grain [1].

The husk is a protective barrier of the grain against external factors, which means that the grain can absorb water more slowly during steeping [1,7]. The percentage of husk in hulled oat grain weight ranged from 20.7% (YO2) to 29.3% (BO) (Table 2). The literature data [49] showed that in naked oat cultivars, the hull is up to 3%, and in malting barley max. up to 15%. On the other hand, in buckwheat grains, the husk constitutes up to 25% [22]. The sieve analysis (Table 2) showed differences in the grain size of individual samples of hulled and naked oats. The oat grains (YO2) were the most even in kernel size (85.6%). The naked oat (NO) grains were the least full, as only 1.6% was retained on the 2.5 mm sieve.

All analyzed cereal samples had normative grain moisture (up to 15%) (Table 2). The total protein content in most of the tested samples of grain intended for malting was comparable to brewing barley and ranged from 10.9 to 11.8% d.m., except samples YO1 and NO with a protein content of 8.8 and 14.4% d.m., respectively (Table 2). These samples were statistically significantly different (*p* ≤ 0.05). Naked oat grain was characterized by too-high protein content (approx. 14.4%) from the point of view of the requirements for brewing barley (below 11.5%). Higher protein content in brewing barley intended for malting is unfavorable, as it often adversely affects the quality parameters of the beer produced [1]. In addition, compared to brewing barley, the assessed oat samples were characterized by a relatively high fat content (average approx. 5.0% d.m.) (Table 2). Klose et al. [40] reported that a higher level of fat in malted grain may be one of the possible reasons for the low foam stability of beer produced from oat malt or with its addition. 

Before the malting process, enzymes in dry grain have limited activity or are still inactive [1]. The studies showed that oat and buckwheat grains had a slight amylolytic activity, as evidenced by the results of diastatic power (4–10 °WK) (Table 2).

### 3.2. Malt and Congress Wort Characteristics

Table 3 summarizes the test results that allow us to characterize the quality of malted grains of oats, buckwheat, and brewing barley, as well as the obtained laboratory wort samples for their use in the production of beer or other fermented beverages. The indicators listed in this table are characterized in the following subchapters.

Table 3 shows the technological quality indicators of the obtained malt samples, such as the efficiency of the malting process and the moisture content of the malt, as well as malt parameters determining the transformation of grain components with the participation of enzymes produced during germination (i.e., extractivity, Kolbach Index, diastatic power, and beta-glucan) and DMS-P content, the level of which in malt depends on the type of germinated grain and the conditions of the malting process (i.e., degree of humidity, germination temperature and time, post-drying temperature). 

Wort parameters such as the content of extract and fermentable carbohydrates, saccharification time of the mash, and the content of soluble nitrogen and free amino nitrogen (converted to d.m. of malt) provide information about the amounts of substances contained in the wort that were dissolved during mashing of malt.

**Figure 1:**
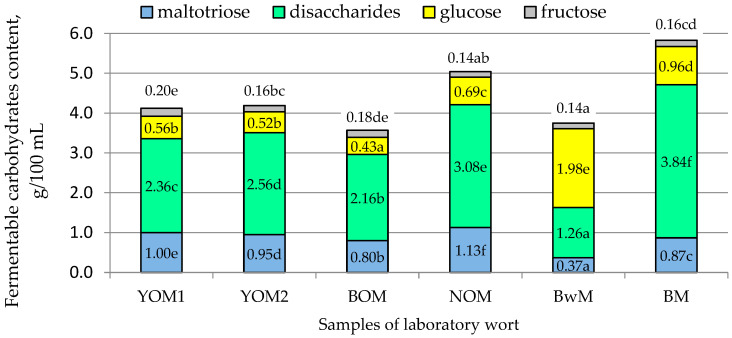
The content of fermentable carbohydrates in the congress wort. Explanations: a, b, c, d, e, f—homogeneous groups at a confidence level of *p* ≤ 0.05; the same letters in rows mean no statistically significant differences between the analyzed values of indicators; sample and indicator codes as in Table 1.

The limit of attenuation of wort determines the amount of alcohol that can be obtained during the fermentation by yeast. In turn, the mash filtration time and wort viscosity indirectly indicate the presence of gel-like substances that extend the wort filtration time and increase its viscosity. Figure 1 shows the content of individual carbohydrate fractions in the wort fermenting by microorganisms such as yeast and/or lactic acid bacteria.

#### 3.2.1. Malting Yield

Evaluation of the malting yield (Table 3) showed that grain mass losses related to grain respiration and germination were about 5% higher for BM and NOM samples than for hulled oat malt samples. The reason for smaller losses in the malting process of hulled oat grains could be the husk, constituting approx. 20–30% of the whole grain (Table 2). Compared to samples with a small share of husk in the grain mass, this could have limited water absorption into the grain and thus slowed down the course of life changes in germinating grains under the assumed conditions of malting time and temperature, recommended for brewing barley [44].

#### 3.2.2. Extractivity, Kolbach Index, and Diastatic Power of Malt

Malt samples produced on a microtechnical scale had normative moisture content (Table 3). Malts used in brewing are dried to a moisture content below 5%, which ensures the durability of the malted grain and stops metabolic processes, including a further increase in enzymatic activity and reducing the possibility of microbial contamination. During storage, malt matures (physical and chemical changes), which facilitates processing in the brewhouse. 

A very important indicator is the content of extract in the dry matter of malt, i.e., its extractability. Results showed that the extractivity of malted grains differed significantly (*p* < 0.05) across the samples (Table 3). In light malts, the extractability should not be less than 79% [1,50]. Among the analyzed samples of oat malts, the NOM sample (79.5% d.m.) showed the highest extractivity, and in the case of hulled oat malts—the extract content of malt ground into flour (2.0 mm) ranged from 51.9 (BOM) to 64.4% d.m. (YOM2). Similar values were obtained by other authors, e.g., Mikyška et al. [12]—59.1% d.m., Kreisz et al. [13]—64.4% d.m., although Pawłowska et al. [48] reported lower values of extractivity (31.2% d.m.). The extractivity of buckwheat malt was 61.8% d.m., and it was consistent with the results obtained by other researchers [11,28]. 

Analysis of the data in Table 3 shows that the smallest amounts of soluble nitrogen (SN) were found in the YOM1 sample (531 mg/100 g d.m.), and the largest—NOM (933 mg/100 g d.m.). Both of these samples were characterized by the highest Kolbach Index (40 and 42%, respectively), which indicates the degree of modification of malt proteins as the ratio of soluble nitrogen (SN) in the wort to total nitrogen in malt. Low-molecular-protein decomposition products, i.e., amino acids present in the wort, are necessary as a nutrient for beer yeast [1,11,16,30]. Among the tested oat malts, the highest amounts of free amino nitrogen (FAN) were found in the laboratory wort with naked oat malt (NOM), while in the remaining samples, the amount of FAN was within the range of 108–134 mg/100 g d.m. of the malt. In the case of buckwheat malt (BwM), the SN content was about 50% lower than in the barley malt (BM) sample, while the FAN concentration was only 1/3 of the amount determined in the barley malt wort. The percentage of FAN in SN in samples of atypical malts ranged from 18.4% (BwM) to 23.0% (YOM1), which should ensure sufficient amino nitrogen in the beer wort for proper yeast growth [1].

Diastatic power (DP) is a measure of the amylolytic activity of malt. Compared to barley malt (BM), oat malts showed low amylase activity, with the highest value of diastatic power in the NOM sample (72 °WK) (Table 3). The tested malt samples did not meet the minimum diastatic power requirements for wheat malt (>87 °WK) [44] and even more so for barley malt (≥240 °WK) [50]. Buckwheat malt (BwM) was characterized by the lowest pool enzymes, as evidenced by the values of diastatic power (30 °WK) and Kolbach Index (25%). Wijngaard et al. [51], in the obtained buckwheat malt with the degree of grain steeping to a humidity of 35, 40, and 45%, obtained values of the Kolbach Index from 23.09% to 24.18%.

#### 3.2.3. Saccharification Rate of Mash and pH Value of Congress Wort

The saccharification is the complete decomposition of liquefied starch by amylolytic enzymes contained in the produced malt to fermentable carbohydrates and dextrins [1,11,52]. The saccharification time of mash, also known as the iodine test, should be negative within 15 min for pale barley malt and within 35 min for dark barley malt [44,50]. The saccharification time of mash from oat malts was longer than that of barley malt and ranged from 10 to 15 min (YOM1 and NOM) to 20 to 25 min (BOM), while the iodine test showed no saccharification of the buckwheat malt mash after 1 h (Table 3). Similar observations are described in the literature [11,13,30,52,53]. 

The literature [11,29,30,54] states that the use of atypical malts for beer production required modification of the mashing process or the addition of enzyme preparations to the mash for greater extraction of protein and carbohydrate substances from malt. For example, Kordialik-Bogacka et al. [30], using enzyme preparations at the oat malt mashing stage, achieved many benefits such as increasing the wort extract and free amino nitrogen, lowering the viscosity of the wort, which translated into faster filtration and a larger volume of the wort. Similar observations were made by Salamon [55] using commercial enzymatic preparations for mashing buckwheat malt to improve the technological parameters of congress wort. In addition, the author showed that the highest concentration of fermentable carbohydrates has been found in the wort produced with cytolytic enzymes.

The pH values of the tested samples were in the range of 5.71–6.10 and differed significantly (Table 3). The pH influences the enzymatic degradation processes during mashing and determines the solubility of proteinaceous substances, hop bitter substances, and the increase in color during wort boiling. Moreover, beer with a high pH shows a tendency towards low physico–chemical stability manifested in the form of turbidity, a consequence of poor protein coagulation in the brewhouse. The pH of the wort in brewing is usually analyzed for the purpose of ascertaining whether the wort pH is optimal for the activity of enzymes present in the barley malts [1]. The pH value of the congress wort should average between 5.6 and 5.9. These pH values were shown by the control sample (BM) and NOM (Table 3).

#### 3.2.4. Wort Viscosity and Time of Filtration of Mash

The viscosities of oat malt wort samples ranged from 1.65 to 1.76 mPa·s, and they were significantly higher than the viscosity of barley wort (1.50 mPa·s) (Table 3). Buckwheat wort was characterized by the highest viscosity (1.90 mPa·s). The viscosities of buckwheat wort determined by Hübner and Arendt [8] ranged from 2.14 to 2.78 mPa·s. 

The filtration time of wort obtained from atypical malts was over 3 h, and only the buckwheat malt mash was filtered for less time (Table 3). According to many researchers [11,30,49,54,56], the presence of beta-glucans or non-starch polysaccharides in the mash could have increased the filtration time of the mash and increased the viscosity of the wort. 

The results of beta-glucan content in the malt samples (Table 3) were significantly lower (0.04–0.10% d.m.) than that of barley malt (0.42% d.m.). In our own research (unpublished data), the determined amounts of beta-glucan in the laboratory wort from oat and buckwheat malt were on the limit of the method’s determination (below 5 mg/L). Therefore, it is assumed that the direct cause of the viscosity of laboratory wort made of atypical malts may be water-soluble arabinoxylans (pentosans), which have similar properties to beta-glucan [56]. Li et al. [57] indicated the existence of statistically significant correlations (*p* ≤ 0.01) between the viscosity of the wort and the amount of arabinoxylans (r = 0.790) and beta-glucans (r = 0.520) contained in the wort, which were extracted during the mashing process. In addition, they found that during the malting of malting barley, an increase in the concentration of water-soluble arabinoxylans was observed from 0.12% to 0.44% d.m. of malt.

#### 3.2.5. Wort Extract, Fermentable Carbohydrates, and Limit of Attenuation of Wort

The extract content is one of the most important parameters of beer wort quality because its amount affects the amount of alcohol in the final product. Data analysis (Table 3) showed that samples of laboratory wort obtained from non-standard malts differed significantly (*p* ≤ 0.05) in terms of the amount of extract and the concentration of fermentable carbohydrates. Barley malt wort (BM) was characterized by the highest extract content (9.17 g/100 mL). The wort sample with NOM was not statistically significantly different from the control sample. The lowest concentration of the extract was determined in the wort with BOM (5.74 g/100 mL). It was shown that oat wort samples’ share of fermentable carbohydrates in the extract was about 57–60%, with the highest amount of carbohydrates in the extract found for barley malt wort (about 64%). 

The largest amounts of fermentable carbohydrates were contained in the control sample (5.83 g/100 mL), followed by malt wort samples: NOM (5.05 g/100 mL), YOM1, and YOM2 (4.10 and 4.20 g/100 mL, respectively). The carbohydrate fraction profile of the analyzed oat malt wort was similar to the barley wort, although the oat wort contained more maltotriose (approx. 10%) and less glucose and disaccharides (Figure 1). No significant differences (*p* > 0.05) were found for the degree of apparent attenuation of the wort of the analyzed samples of hulled oat malts and barley malt, which ranged from 84.8 to 85.9% (Table 3).

The content of the extract in the buckwheat wort was 6.90 g/100 mL (Table 3), and the fermentable carbohydrates present in it accounted for approx. 54%. In the composition of buckwheat wort, more than 50% of glucose was determined, and disaccharides accounted for nearly 1/3 of the fermentable carbohydrate pool (Figure 1). In the studies of Wijngaard and Arendt [28], a similar ratio of glucose in the pool of carbohydrates (52.1%) was obtained in congress wort from buckwheat malt. On the other hand, in wort made from oat and barley malt, the percentage of disaccharides in fermentable carbohydrates was over 60%, and glucose—from 12 to 16% (Figure 1). Very similar results for barley and buckwheat wort were obtained by Deželak et al. [11]. The degree of apparent attenuation of buckwheat wort was 69.2%, and it was approx. 15% less than in the case of samples of barley wort and hulled oat wort samples.

There may be many reasons for the low limit of attenuation of buckwheat wort. According to Kunze [1], beers rich in dextrins are characterized by a low attenuation. Perhaps the content of dextrins in the buckwheat wort was high because there was no saccharification of the mash after 60 min, and the concentration of fermentable carbohydrates in the wort was one of the lowest (3.75 g/100 mL) among the analyzed wort samples (Table 3). In turn, Agu et al. [7] reported that if wort glucose is present in excessive amounts in relation to maltose, some yeast strains may lose the ability to ferment maltose. It is difficult to say unequivocally, but such a situation could have taken place in the case of buckwheat wort, in which the ratio of glucose to maltose was 1.6:1. Perhaps the reason for the low attenuation of buckwheat wort can be found in the insufficient amount of FAN (82 mg/100 g d.m. of malt) (Table 3) because, according to Piddocke et al. [58], the degree of attenuation of the wort generally depends on the use of assimilable nitrogen (FAN) by the yeast. Whereas, Buiatti [59] believes that excess zinc in the wort (above 1 mg/L) is toxic to yeast cells and inhibits enzymes. However, in research by Deželak et al. [11], the brewing yeast *Saccharomyces pastorianus* strain TUM 34/70 used for fermentation utilized about 97% of the total amount of zinc contained in buckwheat wort (1.25 mg/L). Nevertheless, a negative Pearson’s correlation (r = −0.81) was observed between the percentage of assimilated zinc and the ethanol production/extract consumption ratio.

#### 3.2.6. Dimethyl Sulfide Precursors (DMS-P) in Malt

Gas chromatography (GC) analysis revealed that the DMS-P level in barley malt (control sample) was 3.9 mg/kg, and in oat malts, it ranged from 7.4 to 9.5 mg/kg, except for the BOM sample (4.1 mg/kg). Mikyška et al. [12] found a high DMS content in oat beers, reaching almost 500 µg/L, while the DMS concentration in beers obtained from buckwheat and barley malt (1:1) was approximately 40 µg/L. The buckwheat malt sample contained less than 0.1 mg/kg of DMS precursors. Phiarais et al. [16] used buckwheat malt for beer production, which contained 0.5 mg/kg DMS-P.

Dimethyl sulfide (DMS) is a highly volatile sulfur compound that, when detected in beer at levels above 100 µg/L, can give an odor and taste similar to sweet corn or cooked vegetables. According to Kunze [1], to prevent DMS from becoming a problem in the final beer, malt should contain up to 5 mg/kg of DMS precursors (DMS-P). Dimethyl sulfide precursors are formed during the malting process from sulfur amino acids, such as methionine, contained in cereal grains. During mashing and boiling of the wort with hops, thermal processes occur that transform DMS-P into free DMS. Some of the DMS evaporates, and during fermentation, it escapes with carbon dioxide (CO_2_), although some of it remains in the final product [60].

#### 3.2.7. Technological Quality of Oat and Buckwheat Malt and the Possibilities of Their Use in the Production of Fermented Beverages

**Figure 2:**
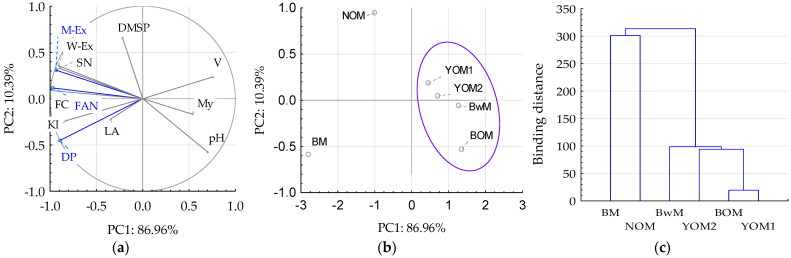
PCA and cluster analysis of technological parameters of malt samples: (**a**) PCA loading plot of two principal components PC1 and PC2, (**b**) score plot presenting analyzed samples in terms of PC1 vs. PC2, (**c**) cluster analysis. Blue lines at (**a**) indicate active data included in the PCA analysis. The blue line on (**b**) concerns the separation of a group of data with a similar effect to the examined indicators. Explanations: sample and indicator codes as in Table 1.

The above-discussed research results regarding the comparison of parameters of oat and buckwheat grains and the parameters of malt and wort samples produced from them indicate limited possibilities of using them as raw materials instead of traditional barley malt, the main raw material for the production of beer. However, in shaping new characteristics of beer, it seems useful to partially replace the traditional raw material with oat and buckwheat grains. In order to make it easier to evaluate and use the obtained research results, principal component analysis and cluster analysis were used (Figure 2). The following indicators were used for analysis: extractivity of malt (M-Ex), free amino nitrogen (FAN) content in malt and diastatic power of malt (DP), which constituted the first principal component of PC1, and wort extract (W-Ex), soluble nitrogen (SN) content in wort, fermentable carbohydrates (FC) content in wort, Kolbach Index (KI), wort viscosity (V), malting yield (My), pH value (pH) of wort, and dimethyl sulfide precursors (DMS-P) in malt, which constituted the second principal component of PC2. The main components (PC1 and PC2) explain 97.35% of the variability in the properties of malt and wort samples. The quality of the tested malt and wort samples was largely influenced by the indicators that are characterized by longer lines from the center of the circle (Figure 2a). Additionally, M-Ex strongly positively correlated with W-Ex, SN, FAN, and FC (in the range of 0.90–0.99) but negatively with My, pH, and V (r = 0.57–0.78). Strong positive correlations also concerned FAN and FC (r = 0.97), as well as with KI (r = 0.87), and pH (r = −0.82). As shown in Figure 2b, the use of various raw materials in the form of oat and buckwheat grains allowed for the division of the obtained data by separating YOM1, YO2, and BwM samples, as well as BOM samples with similar properties and suitability, and NOM and BM samples with significantly different properties. The type of raw material for brewing was also an important factor in data grouping (Figure 2c). 

The assessment of the technological quality of the atypical brewing malt samples obtained in this study showed their non-standard features compared to barley malt (BM), with the exception of the naked oat malt (NOM) sample with similar quality characteristics. Nevertheless, it is possible to obtain gluten-free beers made from 100% oat or buckwheat malt by optimizing the mashing process and/or using commercial enzyme preparations to increase the enzymatic potential of special malts. 

Compared to typical barley beer, beers made of 100% oat malt with the addition of enzyme preparations to the mash, produced by Kordialik-Bogacka et al. [30], contained similar concentrations of ethanol (respectively, 5.25 and 5.32% vol.) and approx. 52% and 34% lower amounts of esters and higher alcohols. In turn, Zdaniewicz et al. [54] prove that the use of 100% oat malt addition in a beer recipe has no negative impact on the beer production process and the quality of the final product with a subtle, grainy flavor. Oat malt beer obtained by Mikyška et al. [12] was hazy, whereas buckwheat beer had unpleasant “non-hoppy” bitterness. Sebestyén et al. [61], from buckwheat wort with 12 °Plato extract, obtained beer with an alcohol content of 2.96% vol. and with a relatively dark color for this type of beer. In the sensory assessment, the beer obtained high scores, and the taste was compared to roasted sunflower seeds and edible chestnuts. Based on the conducted research, Deželak et al. [11] showed that the buckwheat beer production process allows us to obtain a product that can meet the expectations of consumers looking for beer with an original character.

Hassani et al. [62] informed that applying cereal-based media as a delivery vehicle for potential lactic acid bacteria (LAB) and the differences in the bacterial growth patterns between unmalted and malted grains have been widely studied and demonstrated. According to Charalampopoulos et al. [63], the growth of some LAB strains is higher when the fermentation is carried out in malt-based compared to unmalted grains media, due to differences in fermentable carbohydrates and FAN content. Evaluation of cereal-based beverages fermented with probiotic LAB in the study in Salmerón et al. [64] showed that after 8 h of fermentation at 37 °C, the malt beverage inoculated with *Lactobacillus plantarum* had the highest amounts of acetaldehyde and diacetyl, and the greatest amount of lactic acid inoculated with *Lactobacillus acidophilus*. Moreover, it was found that each microorganism develops different flavors in the media tested, and the right combination of microorganisms and substrates could lead to palatable probiotic products.

These and other numerous research results [7,8,9,14,15,16,17,28,34,39,53] regarding the selection of optimal conditions for the malting process of oat and buckwheat grains, and then the modification of the mashing process and the production of beer with 100% atypical malts or with their partial share in the raw material charge, allow concluding that it is possible to obtain special beers classified as styles of the so-called “new-wave”.

### 3.3. Bioactive Compounds and Antioxidant Activity of Malt

Cereal grains and pseudo-cereals, including oats and buckwheat, are a rich source of biologically active substances and ingredients with health-promoting properties [8,14,17]. Health-promoting substances present in cereals include dietary fiber and beta-glucans, vitamins, minerals, and phytochemicals with antioxidant activities, such as polyphenols and carotenoids.

Biotechnological processes, such as the process of malting cereal grains and pseudo-cereals, are promising alternatives for enriching food products with functional bioactive ingredients [62]. 

Table 4 shows the results of the content of lipid pigments, i.e., carotenoids and chlorophylls, and total polyphenols in the obtained atypical malts, and their antioxidant capacity measured by DPPH radical scavenging.

#### 3.3.1. Carotenoids and Chlorophyll Content in Malt

The carotenoids are phytochemicals responsible for the characteristic yellow color of cereal grains. In plants, carotenoids have various functions related to photosynthesis and photoprotection, and they are precursors of phytohormones [65]. Carotenoids occur in small amounts in all anatomical parts of the caryopses, mainly in embryos, and cereals are a good source of them [66]. 

In the buckwheat malt (BwM) sample (Table 4), the highest amounts of carotenoids and chlorophylls were determined, respectively, at 8.73 and 18.83 µg/g d.m., followed by the BOM sample, which contained 4.04 µg/g d.m. carotenoid pigments and 4.36 µg/g d.m. chlorophylls. In turn, the lowest levels of carotenoids were found in the samples YOM2 (1.47 µg/g d.m.) and NOM (1.76 µg/g d.m.). Relatively large amounts of chlorophylls were determined in the YOM2 sample (11.01 µg/g d.m.) compared to the remaining oat malt samples and the control sample (BM).

Ndolo and Beta [67] report that carotenoid pigments occur in chloroplasts in combination with chlorophyll, and they are unevenly distributed in cereal grains. Unfortunately, carotenoids are very sensitive to external factors such as heat, oxygen, light, acids, and oxidative enzymes. The low water content in cereal grains favors their conservation over time. However, during the storage and processing of cereal grains into food products, carotenoid pigments are lost mainly as a result of oxidation and isomerization [68]. Therefore, the malting process contributes to the loss of carotenoids compared to cereal grains before steeping and germination. Similar observations were made by Özcan et al. [69] during malting of barley. 

#### 3.3.2. Total Polyphenol Content in Malt

Polyphenols are a group of compounds that act as natural antioxidants that prevent the occurrence of many lifestyle diseases, such as atherosclerosis, heart attacks, cancers, allergies, and Alzheimer’s disease [21,62]. Many studies show that sprouted cereals contained larger amounts of polyphenolic compounds than grains before germination, which was correlated with an increase in antioxidant capacity [2,21,69,70]. Kaukovirta-Norja et al. [71] reported that phenolic compounds present in oat grain after germination were in the bound form (58%), especially ester (25%) and glycosidic (15%), and only 2–3% were free phenolic acids. Krahl et al. [72] showed that modifying the malting conditions, such as extending the germination time and increasing the process temperature, contributed to an increase in the level of rutin in buckwheat malt.

Similarly to the case of carotenoid pigments and chlorophyll, the highest amounts of total polyphenols were contained in buckwheat malt (646 mg GA/100 g d.m.) (Table 4). This sample differed statistically significantly (*p* ≤ 0.05) compared to the other assessed malt samples. Total polyphenol content (TPC) in hulled oat malts ranged from 215 to 245 mg GA/100 g dm, with the highest TPC determined in the brown hulled malt (BOM) sample. However, the TPC in the naked malt (NOM) and barley malt (BM) samples were similar and amounted to 305 and 342 mg GA/100 g d.m., respectively. Gasiński et al. [17], regardless of the nitrogen fertilization regimes of oat cultivation, in the sample of malt with covered oat cultivar Kozak obtained an average TPC of 190.36 mg GAE/100 g. Samples of naked oat malts (cv. Amant, Maczo, Polar and Siwek) contained higher polyphenol contents (average 254.4 mg GAE/100 g), with the highest polyphenolic compounds determined in samples of oat malt of the Siwek cultivar (average 289.6 mg GAE/100 g), and the least in the malt from cultivar Amant samples (average 223.0 mg GAE/100 g). The authors [17] noticed that the concentration of phenolic compounds in malts increased on average by 466% compared to oat grains; for example, by 325% for the covered oat cultivar Kozak, and for naked oat cultivars: from 428% for Polar to 587% for Siwek. However in the study by Özcan et al. [69], the level of polyphenols in barley malt determined by the HPLC method was 5.9 mg/100 g higher than in the corresponding barley grain (101.9 mg/100 g).

#### 3.3.3. Antioxidant Activity of Malt

According to many authors [2,17,21,69,70], the antioxidant potential depends on the content of total polyphenols and other non-flavonoid phenolic compounds occurring in cereal grains or malt obtained from them. Rasane et al. [37] report that the antioxidant properties of oats are mainly due to the presence of phenolic compounds such as tocopherols, tocotrienols, phytic acid, flavonoids, and non-flavonoid phenolic compounds such as avenanthramides. Buckwheat contains non-flavonoid phenolic compounds, such as rutin, vitexin, and orientin [72].

Antioxidant activity expressed as the ability to scavenge DPPH free radicals in the tested oat malt samples (Table 4) ranged from 1.20 (YOM1) to 2.33 µM Trolox/g d.m. (NOM). Kaukovirta-Norja et al. [71] noticed that grains of gymnosperm varieties (without hull) showed greater antioxidant activity than hulled varieties. Similar observations were made by Gasiński et al. [17]. Furthermore, these authors suggested that compounds present in the oat hull (which is absent in naked oat malts) do not play a significant role in the development of antioxidative potential in the oat malt, because the majority of changes during grain germination occur in the grain embryo, aleurone layer, and endosperm, as reported by Kunze [1]. However, Koren et al. [70], during the malting process of brewing barley, noted the greatest increase in antioxidant activities during the steeping stage. Spring and winter cultivars showed similar trends during steeping and germination, but kilning had different effects on the antioxidant activity of the varieties. The antioxidant activity of malts was always higher than the corresponding barleys.

The ability to scavenge DPPH free radicals in the control sample, i.e., barley malt (BM), was 3.89 µM of Trolox/g d.m. The buckwheat malt (BwM) sample had the highest antioxidant capacity (8.09 µM of Trolox/g d.m.), which contained the highest amounts of compounds with bioactive properties, i.e., total polyphenols, carotenoid pigments, and chlorophylls (Table 4).

#### 3.3.4. Antioxidant Properties of Malt and Directions of Its Use in the Production of Fermented Foods

Malt contains various compounds from grains (endogenous phenolic compounds) or from the malting process (Maillard reaction products) that can play a significant role in malting and brewing through their antioxidative properties. Malt is the main ingredient that contributes to the antioxidant properties of beers; it is estimated that approx. 70–80% of polyphenols in beer come from malt [2,3,70]. Malt antioxidants play an important role in maintaining the beer’s oxidative stability, but they are also important for the prevention of oxidative stress-associated conditions [14,73].

**Figure 3:**
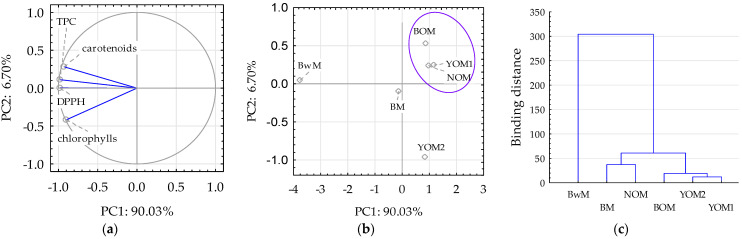
PCA and cluster analysis of the antioxidant potential of malt samples: (**a**) PCA loading plot of two principal components PC1 and PC2, (**b**) score plot presenting analyzed samples in terms of PC1 vs. PC2, (**c**) cluster analysis. Blue lines at (**a**) indicate active data included in the PCA analysis. The blue line on (**b**) concerns the separation of a group of data with a similar effect to the examined indicators. Explanations: sample and indicator codes as in Table 1.

Statistical analysis of the data contained in Table 4 and Figure 3 showed statistically significant (*p* ≤ 0.05) positive Pearson’s correlations between the antioxidant activity (DPPH) of the tested malts and the amount of total polyphenols (r = 0.97), carotenoids (r = 0.86), and chlorophylls (r = 0.78). The results of the study by Ndolo and Beta [67] also showed that positive significant correlations (*p* < 0.05) were found between total carotenoids content, carotenoids analyzed by HPLC (i.e., lutein and zeaxanthin), and DPPH results. The use of oat and buckwheat grains for malt production is interesting in terms of products characterized by an increased content of ingredients with health-promoting properties, such as polyphenol compounds, carotenoids, and chlorophylls, as well as high antioxidant activity, which correlated positively with each other (Figure 3a). Unlike in Figure 2b,c, in Figure 3b,c, the use of raw materials in the form of oat and buckwheat grains allowed for the division of the obtained data by separating samples YOM1, NOM, and BOM with similar properties and suitability, and samples BM, BwM, and YOM2 with significantly different properties.

When producing beer or other malt-based fermented beverages, a decrease in the antioxidant properties of the final products compared to the raw material, i.e., malt, should be taken into account. The literature [70,74] reported that during the beer production process, a reduction in the amount of phenolic compounds and antioxidant activity in the final product was observed. The research of Vanbeneden et al. [75] showed that during the mashing of malt, the level of ferulic acid in the mash increased, while during subsequent stages of beer wort production (i.e., filtration of mash, boiling, cooling), a decrease in its content and an increase in the level of 4-vinylguaiacol, a de-carboxylation product of ferulic acid.

Malted oat grains, especially naked oat malt, can be successfully used for beer production because the quality characteristics assessed in this study are very similar to barley malt. However, it should be borne in mind that the final product may not be an entirely accurate reflection of a typical lager or ale beer. However, by modifying the parameters of malt mashing and wort fermentation, you can receive the so-called “new-wave” product with interesting sensory values.

The high content of bioactive compounds with beneficial effects on human health can undoubtedly be an important criterion for the possibility of using buckwheat malt in brewing. According to Ciocan et al. [76], the production of beer from buckwheat malt with the addition of unmalted buckwheat grain and commercial enzymes enabled the final product to be equivalent in many respects to standard beer. The fermentation profile for buckwheat beer with a higher concentration of glucose was crucial for alcohol and other fermentation products, positively changing its aroma and taste. However, its high content of polyphenolic compounds caused a slightly astringent taste and a spicy and sweet aroma appreciated by tasters who are looking for new sensory experiences.

Considering the fact that malting most cereal plants improves their antioxidant properties, increasing their enzymatic potential and nutritional value, malt can also be successfully used to produce easily digestible food with high nutritional and health-promoting values, and to improve its quality parameters, e.g., in the production of bakery products [77,78,79]. In the study by Molinari et al. [77], gluten-free cookies made with rice flour and buckwheat malt in a 70:30 ratio showed significantly higher content of total phenolic and quercetin, and compared to control cookies, higher anti-oxidant activity, and a lower glycemic index was found (*p* < 0.05). Mäkinen and Arendt [78] report that malts can be used to standardize enzyme levels in baker’s flour because certain levels of amylolytic activities are required for optimal baking performance. Besides, the addition of up to 5% (*w*/*w*) oat malt led to a weakening of the gluten network and subsequent increase in extensibility but, nevertheless, yielded bread with a specific volume higher than the all-wheat control. In turn, results of research by Ninfali et al. [79] showed that replacing 27% wheat flour with malted oat flour allowed for obtaining functional cookies with satisfactory technological properties, high avenanthramide content, and bioaccessibility.

## 4. Conclusions

Products rich in compounds with health-promoting properties and a proper diet can contribute to improving the health of society. Cereals are a source of valuable biologically active ingredients, and malted oat and buckwheat grains can be successfully used to produce gluten-free fermented beverages or products with their addition, e.g., in baking and confectionery. Due to the potential antioxidant properties of polyphenols in typical raw materials used for beer production, it seems necessary to undertake further research to assess gluten-free products fermented with brewer’s yeast and/or lactic acid bacteria (LAB) in terms of antioxidant capacity resulting from the presence of polyphenolic compounds in their composition. In addition, supplementation of wheat flour with malt and/or non-malt flours from the above-mentioned cereals may contribute to improving the baking, nutritional, and functional properties of bread. The obtained results can be the basis for designing fermented foods with health-promoting properties, dedicated to a given disease entity or people who prefer a healthy lifestyle or are looking for nutritionally attractive new products on the food market.

## Figures and Tables

**Table 1:** Sample codes, indicator codes, and photos of malt samples.

Sample Code		Indicator Code			
YO1—hulled yellow oat (cultivar 1) YO2—hulled yellow oat (cultivar 2) BO—hulled brown oat NO—naked oat Bw—buckwheat B—brewing barley		M-Ex—Extractivity (extract content in the malt), % d.m. SN—Soluble nitrogen in the wort, mg/100 g d.m. of the malt FAN—Free amino nitrogen in the wort, mg/100 g d.m. of the malt DP—Diastatic power, °WK (Windisch–Kolbach units) W-Ex—Wort extract, % *w/v* V—Wort viscosity (calculated to 8.6 % *w*/*w*), mPa⋅s FC—Fermentable carbohydrates of wort (calculated), g/100 mL DMS-P—Dimethyl Sulfide Precursors (DMS-P), mg/kg KI—Kolbach Index (calculated), % My—Malting yield, % pH—pH value LA—limit of attenuation of congress wort, %			
YOM1—yellow oat malt (cultivar 1) YOM2—yellow oat malt (cultivar 2) BOM—brown oat malt NOM—naked oat malt BwM—buckwheat malt BM—barley malt					
YOM1	YOM2	BOM	NOM	BwM	BM
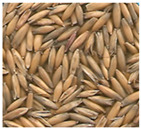	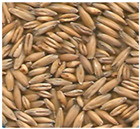	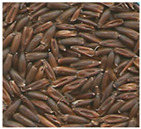	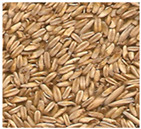	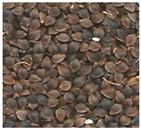	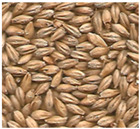

**Table 2:** The selected quality parameters of cereal grains designed for malting.

Quality Parameters	YO1	YO2	BO	NO	Bw	B
Germinative energy after 72 h, %	76 ± 8 ^b^	75 ± 3 ^a,b^	73 ± 3 ^a,b^	80 ± 2 ^b,^*	60 ± 2 ^a^	97 ± 1 ^c,^*
Germination capacity after 120 h, %	79 ± 9 ^a,b,c^	80 ± 5 ^a,b,c^	78 ± 6 ^a,b^	87 ± 2 ^b,c,^*	67 ± 2 ^a^	98 ± 1 ^c,^*
Husk content, %	24.7	20.7	29.3	ns	ns	ns
Sizing of grains (≥2.5 mm), %	34.7	85.6	26.4	1.6 *	ns	98.9 *
Moisture content, %	13.2 ± 0.1 ^d^	13.1 ± 0.1 ^d^	11.4 ± 0.1 ^a^	12.6 ± 0.1 ^c,^*	13.5 ± 0.1 ^e^	12.2 ± 0.1 ^b,^*
Protein content (N × 6.25), % d.m.	8.8 ± 0.1 ^a^	10.9 ± 0.1 ^b^	11.7 ± 0.5 ^b^	14.4 ± 0.2 ^c,^*	11.8 ± 0.1 ^b^	11.3 ± 0,1 ^b,^*
Fat content, % d.m.	3.4 ± 0.1 ^b^	3.9 ± 0.1 ^c^	5.2 ± 0.1 ^d^	7.3 ± 0.1 ^e,^*	2.0 ± 0.1 ^a^	1.7 ± 0.2 ^a,^*
Diastatic power, °WK	9	6	ns	10	4	79

Explanations: ns—not studied, a, b, c, d, e—homogeneous groups at a confidence level of *p* ≤ 0.05; the same letters in rows mean no statistically significant differences between the analyzed values of indicators, *—data published by Salamon [47]; sample and indicator codes as in Table 1.

**Table 3:** The technological quality parameters of malt and congress wort samples.

Quality Parameters	YOM1	YOM2	BOM	NOM	BwM	BM
Malting yield, %	90.4 ± 0.5 ^b^	90.8 ± 1.0 ^b^	90.2 ± 0.1 ^b^	85.0 ± 0.9 ^a^	84.8 ± 0.6 ^a^	85.0 ± 2.1 ^a^
Moisture content, %	4.4 ± 0.1 ^b^	4.7 ± 0.1 ^b,c^	4.5 ± 0.1^b,c^	4.3 ± 0.6 ^b,^*	3.2 ± 0.1 ^a^	5.4 ± 0.1 ^c,^*
Extractivity, % d.m.	62.2 ± 0.2 ^b^	64.4 ± 0.4 ^c^	51.9 ± 0.1 ^a^	79.5 ± 0.2 ^d,^*	61.8 ± 1.0 ^b^	82.8 ± 0.2 ^e,^*
Kolbach Index, %	40 ± 1 ^c^	33 ± 1 ^b^	29 ± 2 ^a,b^	42 ± 1 ^c,^*	25 ± 1 ^a^	53 ± 2 ^d,^*
Soluble nitrogen, mg/100 g d.m.	531 ± 2 ^b^	625 ± 36 ^c^	536 ± 4 ^b^	933 ± 18 ^d,^*	446 ± 19 ^a^	896 ± 14 ^d,^*
Free amino nitrogen, mg/100 g d.m.	122 ± 6 ^a,b^	134 ± 2 ^b^	108 ± 1 ^a,b^	186 ± 18 ^c,^*	82 ± 1 ^a^	219 ± 25 ^c,^*
Diastatic power of malt, °WK	45 ± 1 ^a^	47 ± 3 ^a,b^	50 ± 3 ^a,b^	72 ± 2 ^b,^*	30 ± 1 ^a^	369 ± 15 ^c,^*
Saccharification time of mash, min	10–15	15–20	20–25	10–15 *	>60	<10 *
Time of wort filtration, h	>3	>3	>3	>3 *	>2	1 *
pH	6.02 ± 0.02 ^c,d^	5.98 ± 0.02 ^c^	6.02 ± 0.01 ^c,d^	5.71 ± 0.01 ^a,^*	6.10 ± 0.02 ^d^	5.88 ± 0.02 ^b,^*
Wort viscosity (8.6% *w*/*w*), mPa·s	1.66 ± 0.05 ^b^	1.65 ± 0.04 ^b^	1.76 ± 0.01 ^b^	1.75 ± 0.03 ^b,^*	1.90 ± 0.04 ^c^	1.50 ± 0.04 ^a,^*
Beta-glucan in malt, % d.m.	0.06 ± 0.01 ^a,b^	0.04 ± 0.01 ^a^	0.10 ± 0.01 ^c^	0.06 ± 0.01 ^a,b^	0.06 ± 0.01 ^a,b^	0.42 ± 0.02 ^d^
Wort extract, g/100 mL	6.82 ± 0.07 ^b^	7.29 ± 0.01 ^c^	5.74 ± 0.08 ^a^	8.92 ± 0.03 ^d,^*	6.90 ± 0.09 ^b^	9.17 ± 0.02 ^d,^*
Fermentable carbohydrates of wort, g/100 mL	4.10 ± 0.02 ^c^	4.20 ± 0.01 ^d^	3.56 ± 0.01 ^a^	5.05 ± 0.01 ^e,^*	3.75 ± 0.01 ^b^	5.83 ± 0.04 ^f,^*
Limit of attenuation of wort, %	85.1 ± 0.6 ^c^	84.8 ± 0.6 ^c^	85.0 ± 1.3 ^c^	80.4 ± 2.1 ^b,^*	69.2 ± 0.1 ^a^	85.9 ± 0.6 ^c,^*
DMS-P content in malt, mg/kg	7.4 ± 0.8 ^d^	7.8 ± 0.1 ^d^	4.1 ± 0.1 ^c^	9.5 ± 0.1 ^e^	<0.1 ^a^	3.9 ± 0.1 ^b^

Explanations: a, b, c, d, e, f—homogeneous groups at a confidence level of *p* ≤ 0.05; the same letters in rows mean no statistically significant differences between the analyzed values of indicators; *—data published by Salamon [47]; sample and indicator codes as in Table 1.

**Table 4:** The content of compounds with bioactive properties in malts and antioxidant activity of malts.

Quality Parameters	YOM1	YOM2	BOM	NOM	BwM	BM
Carotenoids content, µg/g d.m.	2.90 ± 0.61 ^a,b^	1.47 ± 0.66 ^a^	4.04 ± 0.48 ^b^	1.76 ± 0.21 ^a,b^	8.73 ± 0.18 ^c^	3.22 ± 1.01 ^a,b^
Chlorophylls content, µg/g d.m.	4.51 ± 0.62 ^a^	11.01 ± 2.03 ^a,b^	4.36 ± 0.43 ^a^	3.65 ± 0.24 ^a^	18.83 ± 7.73 ^b^	8.66 ± 2.11 ^a,b^
Total polyphenol content, mg GA/100 g d.m.	225 ± 8 ^a^	215 ± 11 ^a^	245 ± 11 ^a^	305 ± 12 ^a^	646 ± 93 ^b^	342 ± 4 ^a^
Antioxidant activity (DPPH), µM Trolox/g d.m.	1.20 ± 0.13 ^a^	1.63 ± 0.07 ^a^	1.45 ± 0.32 ^a^	2.33 ± 0.09 ^b^	8.09 ± 0.05 ^d^	3.89 ± 0.18 ^c^

Explanations: a, b, c, d—homogeneous groups at a confidence level of *p* ≤ 0.05; the same letters in rows mean no statistically significant differences between the analyzed values of indicators; sample, and indicator codes as in Table 1.

## Data Availability

The data used to support the findings of this study can be made available by the corresponding author upon request.
